# Tissue Engineering for Massive Bone Defects: The Volumetric Scaling Problem and Strategies to Solve It

**DOI:** 10.3390/bioengineering13070814

**Published:** 2026-07-16

**Authors:** Sedeek Mosaid, Yousif Jihad, Mostafa Jihad, Ashok Marudanayagam, Paul Lee

**Affiliations:** 1 United Lincolnshire Teaching Hospitals NHS Trust, Lincoln LN2 5QY, UK; 2 Lancashire Teaching Hospitals NHS Foundation Trust, Preston PR2 9HT, UK

**Keywords:** massive bone defects, tissue engineering, vascularisation, scaffold design, mPCL-TCP, type-H vessels, sensory innervation, mechanobiology, large-animal models, clinical translation

## Abstract

Massive segmental bone defects present significant challenges in orthopaedic and maxillofacial reconstruction. As defect size increases, the disparity between tissue volume and diffusion-limited biological processes becomes more pronounced. This narrative review analyses distinctions between centimetre-scale defects and conventional fractures and evaluates current tissue-engineering strategies in relation to vascularisation, osteogenesis, mechanical stability, immune response, neural integration, and manufacturability. This review synthesises evidence from scaffold design, cell-based approaches, growth factor delivery, type-H vessel biology, NGF–TrkA signalling, large-animal models, and early clinical translation. Current findings indicate that no single scaffold, cell source, or growth factor can reliably reproduce the coordinated biological and mechanical environment required for durable regeneration of human long bones. The strongest preclinical evidence is derived from ovine tibial models employing medical-grade polycaprolactone/β-tricalcium phosphate composites or mechanobiologically optimised titanium lattices. Human data remain limited to case reports and small early clinical series, including hybrid vascularised flap–scaffold reconstructions. Successful clinical translation will require patient-specific constructs that integrate rapid vascularisation, appropriate load sharing, immune-compatible degradation, infection control, and scalable manufacturing. Until robust comparative clinical evidence emerges, established reconstructive methods will remain the standard of care. Hybrid vascularised scaffold-guided strategies should be regarded as promising translational approaches rather than definitive solutions.

## 1. The Clinical Problem of Massive Bone Defects

Massive bone defects usually happen after high-energy injuries, cancer surgery, aggressive treatment for bone infection or non-union, and severe bone loss during revision joint replacement [1,2]. There is no single agreed-upon definition for critical-size diaphyseal defects. Some commonly used criteria are defects that are several centimetres long, more than twice the bone’s diameter, or involve losing over half of the bone’s outer layer [1,2]. The term ‘massive’ refers to the most severe cases, where rebuilding the bone is especially difficult. In the jaw, defects longer than about 5–6 cm are generally not suitable for non-vascularised grafts, especially if the recipient bed has been exposed to radiation, has scarring, or has poor blood supply. This size limit is a practical guideline, not a strict biological rule [3,4].

The size of the defect is not the only factor to consider when choosing a graft. Blood supply to the area, prior radiation treatment, current or prior infection, soft-tissue quality, fixation options, and the patient’s overall health are all important factors in deciding whether non-vascularised reconstruction is possible. These thresholds can vary depending on the location, fixation method, soft-tissue coverage, and patient factors. A major challenge is that the amount of healthy bone, blood flow, stability, and soft-tissue support required often exceeds what the body or standard implants can provide. Because true segmental defects are especially high-risk, non-union rates from general fracture cases should not be used for these patients [1,2].

Current reconstructive options are effective in certain clinical situations; however, each has limitations that depend on the size of the defect. This review examines strategies for post-traumatic, cancer-related, and infectious diaphysis and mandibular defects. Managing periprosthetic bone loss in revision arthroplasty requires different approaches, such as metallic augments, modular implants, and trabecular metal constructs, which are beyond the scope of this study. Iliac crest autografts provide osteogenic cells, osteoinductive factors, and an osteoconductive matrix that promotes bone healing. However, their use is restricted by the limited amount of graft available, significant donor-site problems, and insufficient strength for unsupported load-bearing diaphyseal reconstruction [2,5]. Structural cortical allografts can offer larger grafts, but they usually incorporate slowly and often incompletely [6,7]. Mechanical weakening and late fatigue fractures are common [7], and infection or immune complications are also significant risks, as shown in large institutional allograft studies [8,9].

Distraction osteogenesis using Ilizarov bone transport can rebuild large segmental defects, usually 6–10 cm, and even longer. This method requires long-term fixation and can lead to complications such as pin-site infections, abnormal bone growth, and delayed or failed union at the docking site [2,10]. Vascularised free fibular transfer provides a long segment of living bone with its own blood supply; however, its initial mechanical strength is limited until union and adaptive hypertrophy occur [11,12]. Custom metallic implants, such as 3D-printed titanium cages and modular tumour-type endoprostheses, are used in some cancer and trauma cases, where filling the space is the main goal. Unlike biologically active methods, these devices replace bone rather than promote its growth, and their success depends on strong fixation and adequate soft-tissue coverage [2,13].

The Masquelet-induced membrane technique uses a polymethylmethacrylate (PMMA) spacer to create a membrane rich in blood vessels and growth factors around a cancellous autograft. This method has been used for very large defects, including some of the largest segmental cases in the literature, as reviewed by Alford et al. [14]. However, it is a two-stage process and is limited by the amount of available graft, fixation, patient biology, and soft tissue coverage. Therefore, no established option consistently and reliably solves this challenge for all defect types, sizes, or patient conditions. Each method has limitations that become more serious with larger defects. Table 1 summarises these options, their mechanisms, practical size limits and main constraints.

Massive bone defects arise in clinical settings that differ substantially in loading environment, vascular bed, soft-tissue envelope, infection risk, fixation strategy, and regulatory pathway; these differences materially affect which reconstructive strategies are feasible and how they should be evaluated. Table 2 makes these distinctions explicit across the common reconstruction contexts. Because the highest-quality experimental and clinical evidence concerns diaphyseal long-bone reconstruction, the review concentrates on this setting, drawing on mandibular and maxillofacial data where directly relevant, while recognising that findings cannot be assumed to transfer uncritically between contexts.

### 1.1. Aim and Objectives of the Review

This narrative review evaluates contemporary tissue engineering strategies for reconstructing massive bone defects. The discussion centres on the volumetric scaling problem, which complicates the healing of large bone defects relative to smaller ones due to increased requirements for vascularity, mechanical stability, bone formation, and scaffold integration.

Tissue-engineering approaches for massive bone defects have been extensively investigated at the materials, cell, and growth factor levels; however, the reasons why individual components consistently fail to produce durable clinical regeneration at the centimetre scale are incompletely understood. This review proposes the volumetric scaling problem—the geometric mismatch between construct volume and the diffusion-limited, temporally constrained biological processes on which regeneration depends—as the organising principle for explaining these failures. Using this principle, the review evaluates contemporary scaffold materials, cellular strategies, vascularisation approaches, immune accommodation, innervation, and mechanobiology in the context of centimetre-scale reconstruction. Evidence from large-animal models and early human clinical cases is analysed, and key knowledge gaps, barriers to clinical translation, and research priorities are identified.

The intended audience comprises clinicians, bioengineers, and translational scientists seeking to understand why effective small-scale bone-repair strategies do not scale to massive defects, and what is required to bridge that gap. The review progresses from the biological and physical principles that define the scaling problem (Section 2), through scaffold design (Section 3), cellular components (Section 4), vascularisation strategies (Section 5), and large-animal and human evidence (Section 6) to persistent translational barriers (Section 7), evidence gaps (Section 8), and conclusions (Section 9). Table 1 summarises the established clinical options against which tissue-engineering strategies should be benchmarked.

The contribution of this review lies not in the individual components, each of which has been reviewed elsewhere, but in applying volumetric scaling as an integrative lens to explain why these components fail to deliver durable regeneration at human scale. Drawing together the mathematics of geometric scaling, diffusion-limited oxygen transport, coupled angiogenesis–osteogenesis, and fixation-dependent load sharing, the review frames the failure of millimetre-scale strategies at the centimetre scale as a coupled multi-system problem rather than a deficiency of any single material or cell type.

### 1.2. Review Methodology

This narrative review was informed by searches of PubMed, Scopus, and Web of Science using terms including ‘massive bone defect’, ‘critical-size bone defect’, ‘segmental bone defect’, ‘tissue engineering’, ‘scaffold’, ‘vascularisation’, ‘bone morphogenetic protein’, ‘mesenchymal stromal cell’, ‘distraction osteogenesis’, and ‘large-animal model’, individually and in combination. Searches focused on English-language publications and were updated to May 2025; as submission occurred subsequently, publications after that date are not comprehensively covered. Studies were included when they reported experimental or clinical evidence directly relevant to scaffold- or cell-based reconstruction of centimetre-scale segmental defects in large-animal or human subjects, or provided foundational mechanistic evidence for the strategies discussed. Consistent with the narrative format, no formal screening algorithm, PRISMA checklist, or systematic quality-grading instrument was applied. The principal limitations of this approach—the potential for selective inclusion of positive findings and the absence of formal evidence-quality assessment—are acknowledged and are revisited in Section 8.

**Figure 1:**
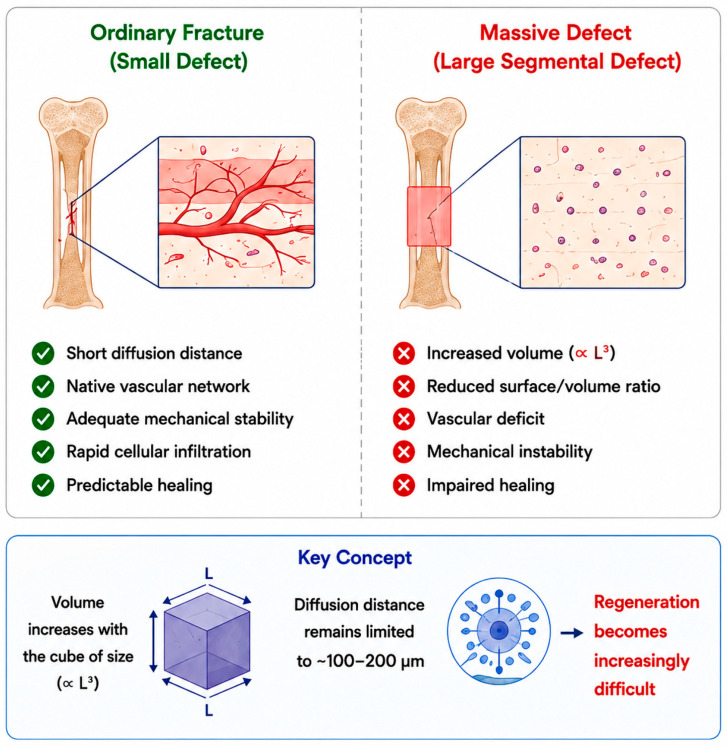
The volumetric scaling problem in bone regeneration. Comparison between ordinary fractures and massive segmental bone defects. As defect dimensions increase, volume rises disproportionately relative to surface area, resulting in increased diffusion distances, impaired vascularisation, mechanical instability, and reduced regenerative capacity.

Figure 1 illustrates the fundamental differences between standard fracture repair and the reconstruction of massive defects, emphasising the significance of the volumetric scaling problem.

## 2. Why Massive Defects Are Biologically Different: The Volumetric Scaling Problem

**Figure 2:**
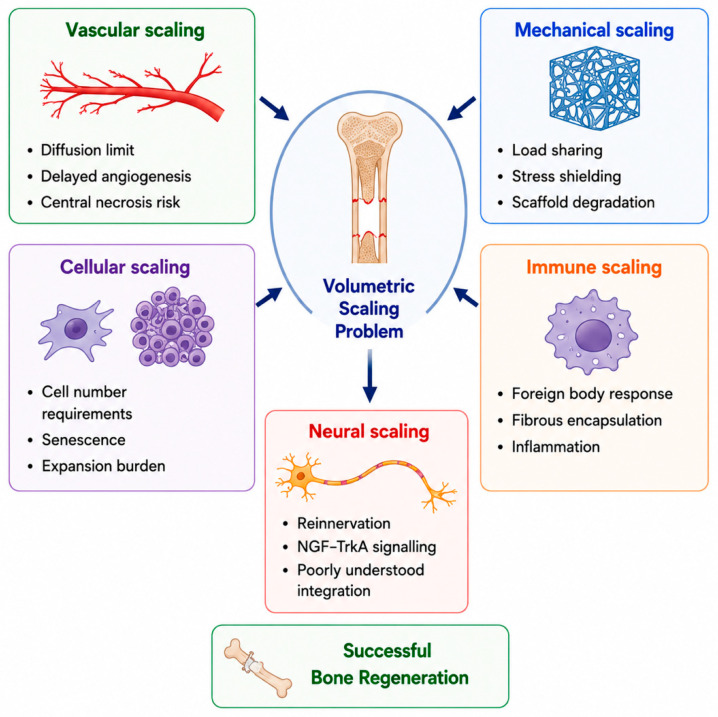
Components of the volumetric scaling problem in massive bone defects. Increasing construct size introduces interconnected vascular, cellular, mechanical, immune, and neural challenges that collectively determine regenerative success.

The primary biological challenges associated with reconstructing large defects arise from the complexities of geometric scaling. Several mechanistic frameworks discussed below—type-H vessel biology, NGF–TrkA innervation signalling, and the human skeletal stem cell phenotype—are presented to convey the biological complexity that centimetre-scale reconstruction must address, and not to imply clinical readiness; none has yet been translated into a validated therapy for human large-bone defects. As tissue size increases, volume expands at a much greater rate than surface area. In tissue-engineered constructs lacking vascularisation, oxygen and nutrient diffusion are typically restricted to approximately 100–200 µm from a capillary, and in some cases up to 300 µm, depending on cellular activity and matrix properties [15,16]. For instance, a 30 mm cube has a volume of approximately 27 cm^3^, which is 1000 times that of a 3 mm cube, while its surface-to-volume ratio is reduced by a factor of 10. At this scale, processes such as vascularisation, cell seeding, mechanical force management, immune acceptance, and, when necessary, nerve regeneration must occur concurrently. Each of these processes is constrained by distinct temporal and biological limitations. Figure 2 illustrates the principal aspects of this scaling challenge.

Vascular coupling. Bone is highly vascularised, and its microvasculature facilitates both nutrient exchange and osteogenic signalling. Type-H endothelial cells, characterised by a CD31ʰⁱEmcnʰⁱ phenotype and initially described in experimental models, are concentrated in the metaphysis and endosteum. These cells connect angiogenesis and osteogenesis through hypoxia-responsive and bidirectional endothelial–osteoprogenitor signalling pathways [17,18]. Their density decreases with age in experimental animal models and is associated with age-related reductions in bone formation [17,18]. Maintenance of the type-H vascular phenotype requires endothelial Notch–DLL4 signalling, which is regulated by Rbpj-dependent pathways. Disruption of this signalling alters the coordination of angiogenesis and osteogenesis in experimental models [18]. Osteoblast-derived SLIT3 targets endothelial ROBO1, and recombinant SLIT3 administration has been shown to enhance fracture healing in mouse models [19]. Preosteoclast-derived platelet-derived growth factor BB (PDGF-BB) also acts as an angiogenic–osteogenic coupling signal on CD31ʰⁱEmcnʰⁱ skeletal endothelium [20]. The selective and safe amplification of these preclinical pathways in human long-bone reconstruction has not yet been established. Furthermore, most mechanistic data on type-H vessels originate from developmental, metaphyseal, ageing, and murine fracture models; direct manipulation or therapeutic application in human diaphyseal segmental defect reconstruction remains unvalidated. Nonetheless, these findings provide a mechanistic basis for the assertion that stable bone regeneration is unlikely to be achieved through unregulated single-factor angiogenic stimulation alone.

Cellular biology. A purified adult human skeletal stem cell population, characterised as PDPN^+^CD146^−^CD73^+^CD164^+^, gives rise to skeletal-lineage-restricted progenitors that generate bone, cartilage, and stromal lineages without adipogenic differentiation [21]; this population is referred to hereafter as human skeletal stem cells (hSSC). This provides a more lineage-restricted framework than the conventional heterogeneous terminology for mesenchymal stem/stromal cells (MSC). This distinction is clinically relevant because bulk mesenchymal stromal cell populations vary in composition and osteogenic potential according to donor factors, harvest sites, and culture conditions. However, prospective clinical isolation, expansion, quality control, and therapeutic delivery of purified hSSC populations for large human bone defects remain experimental and have not yet been demonstrated in adequately powered clinical studies.

Innervation. Bone tissue receives innervation from sensory and autonomic peripheral nerve fibres that express neuropeptides, such as calcitonin gene-related peptide (CGRP), substance P, neuropeptide Y, and vasoactive intestinal peptide [22]. NGF–TrkA signalling within skeletal sensory nerves plays a critical role in adult fracture repair, as chemical or genetic inhibition of TrkA signalling impairs callus vascularisation and ossification in experimental models [23]. Despite these findings, the timing, extent, and functional significance of reinnervation in engineered bone constructs at the centimetre scale remain insufficiently characterised. Most clinically used bone defect constructs do not intentionally replicate organised sensory or autonomic reinnervation. Electroactive scaffolds composed of piezoelectric materials, including PVDF-based polymers, have been investigated for their ability to promote osteogenesis through mechanotransduction [24]. However, intentional strategies to achieve organised sensory or autonomic reinnervation in clinically relevant large bone defects remain in the preclinical stage, and have not been incorporated into approved scaffold systems.

Mechanical scaling. Bone adapts to its mechanical environment in accordance with mechanostat principles: insufficient strain promotes resorption, whereas excessive strain increases the risk of microdamage. The commonly cited microstrain thresholds are approximate and vary depending on the anatomical site, tissue quality, loading mode, and biological context [25]. Composite polymeric scaffolds typically possess elastic moduli lower than those of cortical bone, resulting in a limited initial load-bearing capacity during diaphyseal reconstruction. Consequently, supplementary fixation is generally required throughout the consolidation phase [26]. Triply periodic minimal surface (TPMS) lattice architectures, such as gyroid and Schwarz-type designs, enable the effective modulus and permeability of metallic scaffolds to be adjusted by varying porosity, unit-cell geometry, and strut thickness, while maintaining interconnected pore networks [27]. Designs optimised solely for initial stiffness may become mechanically suboptimal as polymeric scaffolds degrade and the load-sharing environment changes over time. Therefore, mechanobiological scaffold design, including strain-tuned structural optimisation, as demonstrated in vivo by Pobloth et al. [28], is key for achieving predictable regeneration outcomes.

Immune scaling. As the construct volume increases, the host response to foreign materials becomes more significant and is affected by factors such as surface area, surface chemistry, topography, degradation products, local motion, microbial contamination, and patient-specific variables [29]. Persistent macrophage-driven inflammation can lead to fibrous encapsulation, while acidic degradation products from hydrolytic polymers, such as PLA and PLGA, may further impair the local regenerative environment at the implant site. Consequently, contemporary scaffold design seeks to encourage pro-regenerative macrophage phenotypes by controlling the surface topography, ion-release profiles, and surface functionalisation. The conventional classification of macrophages into pro-inflammatory (M1) and pro-regenerative (M2) phenotypes should be regarded as a simplified framework rather than a strict binary classification [30].

## 3. Scaffolds for Centimetre-Scale Reconstruction

Scaffolds for large-scale defects must support normal body loads, resist cyclic fatigue loading, allow blood vessels to grow into them, facilitate nerve connections when needed, manage the body’s immune response, and degrade at a rate that matches new bone growth. Currently, no single material type meets all these requirements (Table 3).

Calcium phosphate ceramics are chemically similar to the minerals found in natural bones. Hydroxyapatite (HA) supports bone growth but can break down slowly or remain in the body for an extended period. In contrast, β-tricalcium phosphate (β-TCP) degrades more rapidly, and its properties depend on factors such as porosity, crystallinity, and preparation method [31]. Mixtures of HA and β-TCP, typically in ratios of 60:40 to 80:20, are commonly used as bone-graft substitutes; however, the optimal ratio for larger repairs depends on the location and clinical need [31]. Bioactive glasses, such as Hench’s 45S5 Bioglass, release ions that help bone grow; however, their brittleness and manufacturing challenges limit their use for large, load-bearing repairs [32]. Polycaprolactone (PCL) slowly degrades over several years and is not sufficiently strong enough on its own. Polylactic acid (PLA) and poly(lactic-co-glycolic acid) (PLGA) degrade more rapidly and can release acids, which is important for larger implants [26].

Combining thermoplastics, such as PCL or PLA, with ceramics, such as β-TCP or HA, can improve printability, support bone growth, and control scaffold degradation rate [33]. The medical-grade polycaprolactone/β-TCP composite (mPCL/β-TCP) is one of the most studied polymer–ceramic combinations in sheep tibial defect models [34,35]. Although its stiffness is still much lower than that of real bone, additional fixation can provide the support needed during healing [26,34,35].

For clinical use, the scaffold’s structure is as important as its material. Pore sizes of 300–500 µm are often recommended for bone growth; however, the optimal size depends on the material, its porosity, cell type, extent to which blood vessels are encouraged to grow, and mechanical environment [36,37]. Pores smaller than 100 µm can help cells attach, but may not allow blood vessels to grow. Larger pores allow better blood vessel growth but can weaken the scaffold if there are too many pores [36,37]. Murphy et al. found that 325 µm was the best average pore size for collagen-glycosaminoglycan scaffolds, demonstrating that the ideal pore size depends on the specific scaffold [38].

In addition to porosity, the overall scaffold design can be carefully planned. Zhang et al. created 3D-printed bioceramic scaffolds that mimic Haversian bone, with channels that facilitate the delivery of different cell types, support the growth of blood vessels, and improve bone healing in large rabbit bone defects [39]. Pobloth et al. have shown that titanium mesh scaffolds, fabricated by selective laser melting and designed for optimal mechanical conditions, reduce stress shielding and promote bone formation in 4 cm sheep tibial defects [28].

**Figure 3:**
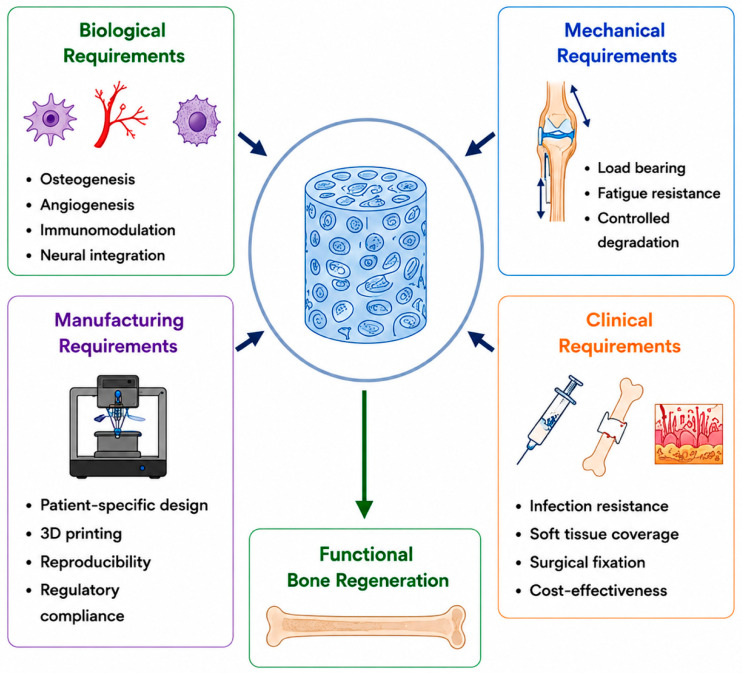
Requirements for successful tissue-engineered reconstruction of massive bone defects. Effective regeneration requires integration of biological, mechanical, manufacturing, and clinical considerations within a unified scaffold-based strategy.

There are two main design principles: the scaffold structure should facilitate the growth of blood vessels into its centre, and its shape should be planned using mechanical and biological principles rather than trial and error. Neural integration is becoming increasingly important, but the intentional restoration of nerves during large bone repairs remains under investigation. Choosing which cells to add to these scaffolds presents more biological and manufacturing challenges, which will be covered in the next section. Figure 3 summarises the main biological, mechanical, manufacturing, and clinical requirements for successful large-scale bone repair.

**Table 3:** Representative scaffold materials and their behaviour at the centimetre scale. Values are qualitative descriptors; actual properties vary substantially with porosity, architecture, testing mode, and manufacturing method. Dense-material benchmarks should not be applied to porous scaffolds. Native bone values are design-context benchmarks. For quantitative design data, see Rho et al. [40] and the source publications.

Material Class	Compressive Performance at cm-Scale (Qualitative)	Stiffness (Relative to Cortical Bone)	In Vivo Resorption Window	Principal Limitation at the cm-Scale	Ref.
Stoichiometric hydroxyapatite (HA)	Very high in dense form; brittle; greatly reduced in porous scaffolds	Very high in dense form; substantially reduced with porosity	Slowly resorbing or relatively persistent (years)	Brittle; limited remodelling; ceramic strut fatigue	[31,40]
β-Tricalcium phosphate (β-TCP)	Moderate in dense form; highly porosity-dependent	Moderate to high in dense form; substantially lower in porous scaffolds	6–18 months (porosity-dependent)	Loss of mechanical competence during resorption	[31]
Biphasic CaP (HA:β-TCP 60:40 to 80:20)	Moderately dense; tunable via HA:β-TCP ratio and porosity	Variable; depends on phase ratio, porosity, and processing	Tunable (months–years) via HA:β-TCP ratio	Optimal HA:β-TCP ratio contested across anatomical sites and indications	[31]
45S5/13-93 bioactive glass	High in dense form; greatly reduced in porous scaffolds	High in dense form (comparable to cortical bone); markedly lower in porous form	Weeks to months	Intrinsic brittleness; processing constraints for load-bearing cm-scale constructs	[32,41]
Polycaprolactone (PCL)	Low; insufficient for unsupported load-bearing	Very low; well below cortical bone	~2–4 years	Bioinert; low strength without ceramic phase	[26]
PLA/PLGA copolymers	Low to moderate; architecture-dependent	Low; decreases as degradation proceeds	1–12 months (composition-dependent)	Bulk-hydrolytic acidic by-products in large volumes	[26]
mPCL/β-TCP composite (80:20 wt%)	Low; supplementary fixation required	Very low; well below cortical bone; fixation-dependent construct	2–4 years (PCL-rate-limited)	Extensively evaluated in cm-scale ovine studies; modulus is still well below the cortical bone	[34,35]
Selective-laser-melted Ti alloy TPMS lattice	Moderate to high; highly architecture-dependent *	Tunable by porosity and unit-cell design; can approach the cortical bone range *	Non-resorbing	Permanent implant; modulus tunable, but does not remodel	[27,28]
Native cortical bone	100–230 MPa (design benchmark)	7–30 GPa (design benchmark)	—	(benchmark for diaphyseal load-bearing design)	[40]
Native trabecular bone	2–12 MPa (design benchmark)	0.1–5 GPa (design benchmark)	—	(benchmark for metaphyseal/cancellous design)	[40]

* Effective values for porous Ti TPMS lattice constructs depend on strut thickness, unit-cell topology, porosity, and testing mode; bulk alloy values are substantially higher. For quantitative reference ranges of dense and porous scaffold materials, see Rho et al. [40] and Rahaman et al. [41].

The distinction between dense-material benchmarks and porous construct-level performance is clinically critical and merits explicit quantification. In dense sintered form, stoichiometric hydroxyapatite achieves compressive strengths of approximately 100–900 MPa and elastic moduli of 70–120 GPa; at clinically relevant porosities (≥40%), however, hydroxyapatite scaffolds typically demonstrate compressive strengths of 2–40 MPa and elastic moduli of 0.5–8 GPa [40]. β-Tricalcium phosphate scaffolds at comparable porosities show moduli substantially below 5 GPa, and bioactive-glass scaffolds at ≥40% porosity typically achieve 5–50 MPa compressive strength with moduli of 0.5–3 GPa [41]. Medical-grade PCL/β-TCP composites at clinically evaluated architectures exhibit compressive strengths well below those of cortical bone and therefore require supplementary fixation throughout consolidation [34,35]. Porous titanium TPMS lattices can be tuned across a substantially wider stiffness range by adjusting unit-cell geometry and porosity, potentially approaching values relevant to load-bearing diaphyseal reconstruction, but do not resorb or remodel [27,28]. All figures are strongly architecture-dependent and should be validated against the specific fabrication parameters of any given construct.

## 4. Cellular Components at a Clinically Relevant Scale

The selection of cellular components is limited by the substantial number of cells required for clinically relevant defects. Bone marrow-derived MSCs (BM-MSCs) represent the most extensively characterised population, as demonstrated by an early clinical report involving three patients with long-bone reconstruction in diaphyseal defects, which provided the first human evidence for this approach [42]. Nevertheless, the BM-MSC yield is inherently low, the harvesting procedure is invasive, and extended in vitro expansion reduces both the proliferative and osteogenic potential [43]. Generating the cell numbers necessary for centimetre-scale constructs typically necessitates multiple rounds of ex vivo expansion, during which replicative senescence progressively diminishes proliferative capacity and osteogenic differentiation. This limitation intensifies as the defect volume increases, complicating direct comparisons between donor sources with varying harvested cell densities [43,44].

Adipose-derived stromal cells (ASCs) offer practical advantages, as liposuction yields substantially higher initial cell numbers than does iliac crest marrow aspiration. However, yield estimates vary depending on the harvest method, isolation protocol and culture conditions [43,45]. The relative superiority of BM-MSCs versus ASCs for centimetre-scale bone reconstruction remains unresolved as adequately powered head-to-head comparative trials are lacking [46].

**Figure 4:**
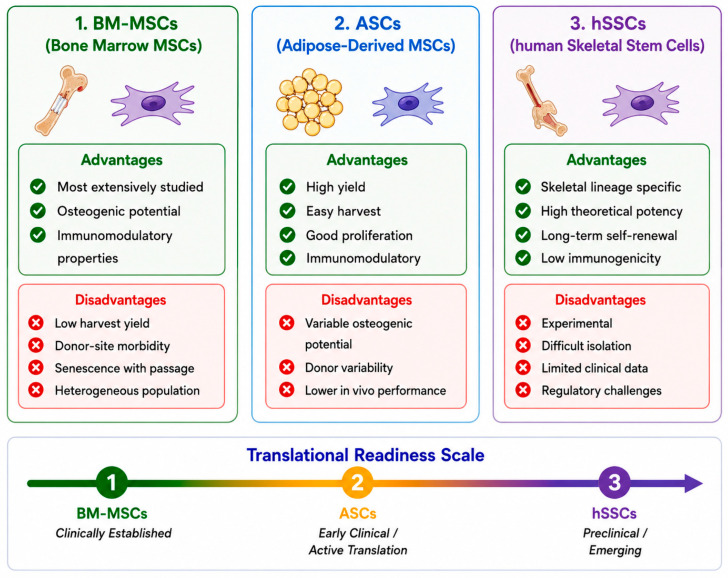
Comparison of cellular sources for bone tissue engineering. Advantages, disadvantages, and translational readiness of bone marrow-derived mesenchymal stromal cells (BM-MSCs), adipose-derived stromal cells (ASCs), and human skeletal stem cells (hSSCs).

The purified PDPN^+^CD146^−^CD73^+^CD164^+^ human skeletal stem cell population offers a more lineage-restricted therapeutic alternative without adipogenic potential; however, its clinical translation is still in its early stages [21]. Figure 4 compares the principal cellular populations currently being investigated for bone tissue engineering.

Periosteal-derived progenitor cells are another source of osteogenic cells and are especially relevant to scaffold-guided methods that incorporate periosteal tissue transfer as a built-in vascular and biological component of the construct. Induced pluripotent stem cells (iPSCs), first derived from mouse fibroblasts using the Yamanaka factors, provide researchers with a means to generate expandable, patient-specific osteogenic progenitors [47]. When these iPSC-derived progenitors are placed on decellularised bovine bone scaffolds, they can form mineralised bone-like structures in the laboratory [48].

Generating patient-specific cells that meet good manufacturing practice (GMP) standards remains technically challenging and time-consuming. Therefore, it is difficult to use these methods for urgent trauma or cancer-related bone reconstruction. Consequently, iPSC-derived bone constructs are not yet used in routine clinical practice. In addition to timing issues, there are concerns about the remaining undifferentiated cells and their potential to cause tumours [49].

There are also regulatory challenges in demonstrating that each batch of cells meets GMP standards, which further slows progress. These issues collectively slow clinical translation, even as preclinical research advances [49]. In large constructs, cell survival and bone formation depend heavily on the speed and completeness of blood vessel formation.

## 5. Vascularisation as a Central Rate-Limiting Barrier in Massive Defect Reconstruction

**Figure 5:**
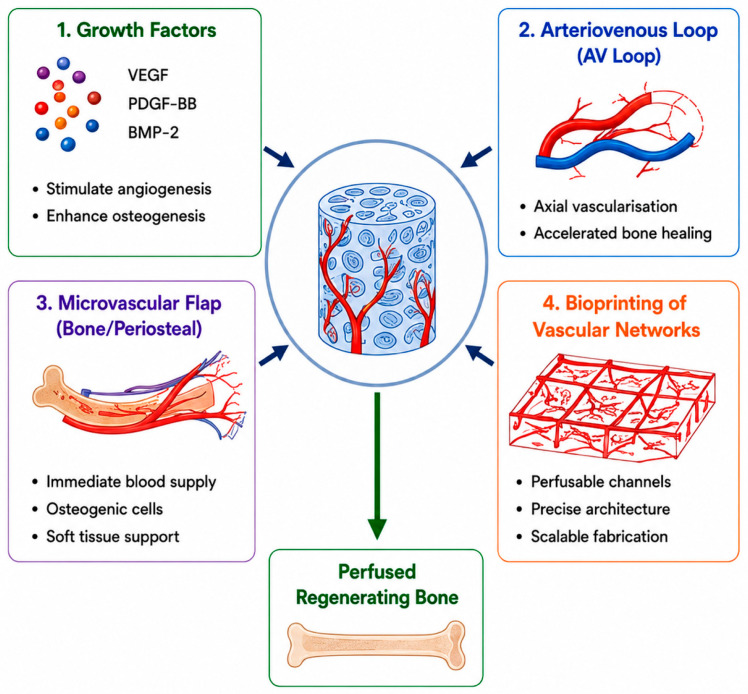
Strategies to overcome vascular limitations in massive bone defect reconstruction. Current approaches include growth factor delivery, arteriovenous loop vascularisation, microvascular flap integration, and bioprinted vascular networks.

Vascularisation remains a key challenge in reconstructing bone at the centimetre scale [15,16]. Strategies are usually grouped into intrinsic, in which constructs are designed to attract the body’s own blood vessels, and extrinsic, in which constructs are pre-vascularised in the lab or in the body before implantation. Figure 5 shows the main approaches used to address this issue.

Growth factor delivery. Researchers have tested the delivery of vascular endothelial growth factor (VEGF) and bone morphogenetic protein-2 (BMP-2), either sequentially or together, from porous scaffolds in preclinical models. This approach can aid bone regeneration in certain situations. However, in real bone defects, the effects are often weaker than in artificial settings and depend heavily on the dose, timing, delivery method, and defect location [50].

Matrix-binding strategies, such as using fibronectin domains to present growth factors, can help retain growth factors and reduce the dose required for them to be effective [51]. Concerns have been raised regarding the safety of recombinant BMP-2 in spinal fusion. A 2011 review found that the rate of adverse events ranged from 10 to 50 per cent, depending on the surgical method, which is much higher than rates reported in some early industry-sponsored studies. The review also listed complications such as heterotopic bone growth, retrograde ejaculation, and cervical dysphagia [52].

Vascularised flap integration. The integration of tissue-engineered constructs with microvascular flaps facilitates the immediate establishment of an axial vascular pedicle for reconstructive procedures. Kneser and colleagues described arteriovenous loop-based axial vascularisation of osteoconductive scaffold compartments before implantation [53]. This approach was applied clinically in mandibular reconstruction by Warnke et al., who utilised a titanium mesh loaded with BMP-7, bovine bone mineral (Bio-Oss), and patient bone marrow, matured in a latissimus dorsi pouch, and subsequently transferred with its vascular pedicle [54]. Similarly, Mesimäki et al. reported maxillary reconstruction using autologous ASCs combined with β-tricalcium phosphate (β-TCP) in an ectopic in vivo bioreactor before microvascular transfer [55].

Bioprinting. Three-dimensional bioprinting enables the fabrication of patient-specific constructs with embedded channels that enhance nutrient transport and address diffusion limitations [56]. Extrusion-based systems, such as ITOP, can produce human-scale constructs with sufficient structural integrity and microchannel architectures to support cell survival within larger constructs [56]. Nevertheless, this technology encounters fundamental engineering trade-offs. Achieving printing resolutions that replicate capillary-scale architecture is constrained by the viscosity requirements of hydrogel bioinks, the need for structural integrity in load-bearing constructs, and the throughput necessary for centimetre-scale fabrication.

Typical extrusion-bioprinted channels are substantially larger, often by one to two orders of magnitude, than native capillary lumina (approximately 5–10 µm in diameter) [15,56]. As a result, these channels function as perfusable conduits rather than forming a complete capillary bed. Functional microvascularisation remains dependent on endothelialisation, inosculation with host vessels, and subsequent remodelling—processes that occur over weeks to months and may not meet the metabolic demands of the implanted cellular content [15,56].

## 6. Large-Animal Validation and Clinical Translation

The ovine tibial segmental defect model is one of the most commonly used large-animal models for centimetre-scale bone reconstruction (Table 4). In this model, a 3 cm mid-diaphyseal defect is stabilised with a locking compression plate, thereby creating a consistent, critical-sized non-union in untreated controls [34]. Reichert et al. found that mPCL-TCP scaffolds containing 3.5 mg rhBMP-7 resulted in bridging by three months and robust regeneration at the 12-month endpoint. These results were similar to those observed with autografts and better than those observed with mPCL-TCP alone or mPCL-TCP combined with BM-MSCs [34].

Cipitria et al. later showed that lowering the rhBMP-7 dose from 3.5 mg to 1.75 mg produced similar results in the same ovine tibial model [35]. In another study, Pobloth et al. used titanium mesh scaffolds designed to achieve optimal mechanical properties in 4 cm ovine tibial defects. The less-stiff scaffold enabled earlier defect bridging and greater endochondral bone formation at 24 weeks than the stiffer scaffold, supporting the idea of strain-tuned mechanobiology [28].

An earlier study by Petite et al., using an ovine metatarsal model, showed that coralline scaffolds made from coral-derived calcium carbonate, when seeded with expanded BM-MSCs, resulted in greater bone formation than scaffolds alone or scaffolds combined with fresh bone marrow [57].

The regenerative matching axial vascularisation (RMAV) method combines a 3D-printed mPCL-TCP scaffold with an autologous corticoperiosteal flap, providing both a vascular pedicle and periosteal progenitor cells. Sparks et al. developed a preclinical and translational basis for RMAV, which was later used by Castrisos et al. in a clinical setting [58,59]. In preclinical tests, RMAV was used in ovine tibial defects of approximately 9.5 cm^3^ (M-size) and a larger 19 cm^3^ pilot defect (XL pilot), showing regeneration similar to autograft and better than scaffold alone.

The technique was also used in a 27-year-old patient with a 36 cm intercalary tibial defect following osteomyelitis, resulting in weight-bearing radiographic consolidation, as reported by the authors [58,59]. This RMAV case adds to the early clinical literature on scaffold-guided regeneration with vascularised tissue transfer and similar long bone treatments [60].

These reports are among the first to demonstrate the direct clinical use of centimetre-scale, load-bearing, scaffold-guided reconstruction in human long bones. However, current clinical evidence is limited to case reports, first-in-human studies, and early cohort follow-ups rather than randomised comparative trials.

## 7. Persistent Barriers Unique to Large-Scale Defects

Eight interconnected barriers continue to separate centimetre-scale tissue engineering from the clinical standard of care.

Vascularisation kinetics. Centimetre-scale constructs depend on blood vessel growth from the host tissue; however, this process is often too slow to support the metabolic needs of the central tissue. Consequently, there is a higher risk of central necrosis unless vascularisation is accelerated or already present [15,16]. Hybrid approaches that combine intrinsic and extrinsic vascularisation have not yet been directly compared in large-animal trials with sufficient statistical power.

Mechanical adequacy. Achieving sufficient mechanical strength under normal body loads remains a challenge. Most hydrogel-based, highly porous scaffolds cannot initially support their own weight. In contrast, designs that focus only on initial stiffness may lose strength as they degrade and the load-sharing changes. Modulus-matched lattice designs, such as TPMS structures and metallic scaffolds optimised for mechanical performance, help address these problems by leveraging computational design and adjusting the structure to handle strain [27,28].

Immune accommodation. As the size of the construct increases, the body’s response to foreign material becomes more important and is influenced by factors such as material chemistry, surface texture, breakdown products, construct movement, infection, and patient-specific differences. Therefore, scaffold design should focus on encouraging a regenerative response in the host rather than relying solely on drugs to suppress the immune system [29,30].

Sensory and autonomic reinnervation. Organised neural integration of large bone defect constructs remains poorly understood clinically. Although new preclinical evidence links NGF–TrkA sensory signalling to callus vascularisation and bone formation [22,23], no approved scaffold system currently includes planned reinnervation strategies, and this aspect is missing from current regulatory guidelines for combination products.

Biological envelope and clinical scalability. The Masquelet-induced membrane represents a clinically relevant model of the biological envelope that scaffold-guided regeneration must replicate or replace. This membrane is highly vascularised, contains growth factors, and promotes osteogenic activity [14]. It may partially reproduce several in vivo functions that tissue-engineered constructs aim to achieve. In a retrospective cohort study, Lodewijks et al. [61] evaluated 10 patients (tibia *n* = 7, femur *n* = 3) with a median segmental defect of 10.5 cm (IQR 3.25), treated using the induced-membrane technique combined with 3D-printed PCL/TCP scaffolds. Complications occurred in seven of the 10 patients, all of whom required additional surgery. Five patients underwent complete revision with a newly induced-membrane procedure, with infection identified as the primary cause.

These findings indicate that envelope biology and vascular supply, rather than scaffold composition alone, are critical determinants of the size of reconstructible defects. Scaffold-guided reconstruction in humans remains technically challenging despite promising preclinical data. Moreover, direct comparative clinical evidence at the required scale is limited [14].

Infection. Infection is not merely a complication of massive defect reconstruction but is often the primary driver [1,2]. In cases of post-traumatic and osteomyelitis-related defects, previously infected or contaminated sites generally require staged debridement and infection control before the safe application of scaffold-based strategies. Active or recurrent infection was identified as the leading cause of failure and surgical revision in the Lodewijks et al. clinical cohort [61]. Although scaffold-guided regeneration trials have largely excluded infected defect beds, this patient population represents the group in which alternatives to autografts and distraction osteogenesis are most urgently required.

Staged infection–eradication protocols, such as the use of antibiotic-impregnated cement spacers in the initial stage of the Masquelet technique, have demonstrated clinical feasibility [14]. However, the systematic integration of infection control with next-generation bioactive scaffold components, including antimicrobial surface functionalisation and local antibiotic-eluting modifications, remains unaddressed in current scaffold-guided regeneration research and constitutes a significant future design priority.

Host- and patient-related factors. Comorbidities such as diabetes mellitus, tobacco use, prior radiotherapy, systemic immunosuppression, malnutrition, and inflammatory disease directly impair the vascular, cellular, and immunological responses required for scaffold-guided regeneration [1,2]. These conditions affect recipient-bed vascularity, soft-tissue envelope quality, infection susceptibility, and fixation durability, which are key determinants of the clinical feasibility of non-vascularised reconstruction [1,2].

Preclinical large-animal models generally use young, healthy, standardised animals in controlled environments. Consequently, the adverse effects of these host factors are systematically absent from the experimental evidence base summarised in Table 4. This omission represents a significant and underexplored contributor to translational failure that warrants consideration in future clinical trial design.

Regulatory and manufacturing complexities. The regulatory pathway for integrated scaffold–cell–factor constructs is highly complex, particularly for GMP-compliant, patient-specific production within the clinical timelines required for trauma or oncology applications. Classification depends on composition, degree of manipulation, and primary mode of action; products may be designated as advanced therapy, biologic, device, or combination products, each subject to distinct evidentiary requirements across jurisdictions [62,63].

Regulatory frameworks in this field have advanced beyond those described in previous studies [62,63]. Applicants should consult jurisdiction-specific guidance from the European Medicines Agency (EMA) Committee for Advanced Therapies (CAT), the United States Food and Drug Administration (FDA) Center for Biologics Evaluation and Research (CBER) Office of Therapeutic Products (OTP; formerly the Office of Tissues and Advanced Therapies), and, where relevant, the FDA Office of Combination Products, according to the primary mode of action of the product. Emerging approaches, such as the woven-bone organoid system, described by Akiva et al. [64], continue to face unresolved immunological, lot-release, manufacturing, and regulatory challenges that are broadly relevant to advanced therapy constructs at the clinical scale.

For combination products incorporating living cellular components, regulatory agencies typically require an evidence package spanning several domains: batch-level sterility, bioburden, and endotoxin testing; defined cell identity, viability, purity, and potency assays underpinning GMP release criteria (extending the lot-release considerations noted above); mechanical testing before and after sterilisation together with degradation profiling under simulated physiological conditions; immunogenicity characterisation of both scaffold and cellular components; infection-control evidence where degradable components may harbour biofilm; and long-term safety surveillance, including the tumorigenicity monitoring already noted for expanded or iPSC-derived populations. Product classification itself—as a tissue-engineered product, combined advanced therapy medicinal product, or device–biologic combination—depends on the primary mode of action and follows established regulatory criteria [65]. Patient-specific constructs pose an additional challenge, since release criteria must be established for individually fabricated products, limiting the applicability of conventional process-validation approaches. Developers proposing first-in-human applications are therefore advised to seek early scientific advice from the EMA Committee for Advanced Therapies or the FDA CBER Office of Therapeutic Products to agree on the required data package before committing to a development pathway.

## 8. Knowledge Gaps, Contradictions, and Evidence Strength

Several apparent contradictions in the centimetre-scale reconstruction literature reflect genuine evidence gaps rather than methodological flaws. The optimal scaffold pore size remains unresolved because it depends on variables such as cell type, scaffold composition, porosity, vascularisation strategy, and the mechanical environment of the defect. Karageorgiou and Kaplan identified the 300–500 µm range as broadly favourable for bone regeneration, while Murphy and colleagues reported approximately 325 µm as the most favourable mean pore size in collagen-glycosaminoglycan scaffolds [36,37,38]. The optimal hydroxyapatite-to-beta-tricalcium phosphate (HA:β-TCP) ratio is also anatomically and biologically context dependent [31]. Furthermore, comparative clinical evidence is currently insufficient to support the selection of adult MSC sources, such as bone marrow-derived MSCs (BM-MSCs) versus ASCs, for centimetre-scale defects [43,45]. A further critical limitation concerns the systematic under-representation of failed translations and negative results. The studies summarised in the preceding sections represent cases in which constructs were applied and measurable outcomes reported; abandoned clinical applications, scaffold failures requiring revision without reported success, and preclinical models in which tissue-engineering strategies proved inferior to conventional reconstruction are substantially less likely to be published. This publication bias, which is well documented across orthopaedic and surgical research [66], risks inflating the apparent clinical readiness of scaffold-guided reconstruction. No adequately powered randomised trial has directly compared any tissue-engineering strategy with autograft, distraction osteogenesis, or vascularised fibular transfer, so the current evidence base cannot support guideline-level recommendations. Future first-in-human studies should be prospectively registered and should report standardised outcomes—including CT-based bridging scores, patient-reported outcome measures, and complete complication data—under pre-specified analysis plans so that unsuccessful as well as successful applications enter the literature.

Three widely cited mechanistic frameworks exhibit significant evidence gaps that are not consistently acknowledged in the literature. The type-H vessel paradigm is primarily supported by murine models, and only limited but increasing evidence suggests that human metaphyses contain CD31ʰⁱEmcnʰⁱ endothelial populations [67]. The clinical relevance of the type-H vessel paradigm to long-bone reconstruction remains inferred rather than empirically demonstrated. The clinical translation of induced pluripotent stem cell (iPSC)-derived osteoprogenitors has not yet been achieved due to unresolved regulatory, GMP-directed differentiation, and immunogenicity concerns [49]. Additionally, common extrusion-bioprinted channels are substantially larger, often by one to two orders of magnitude, than native capillary lumina (approximately 5–10 µm in diameter). This size mismatch suggests that current bioprinted ‘vascular’ channels more closely resemble larger conduits than capillary beds [56].

**Figure 6:**
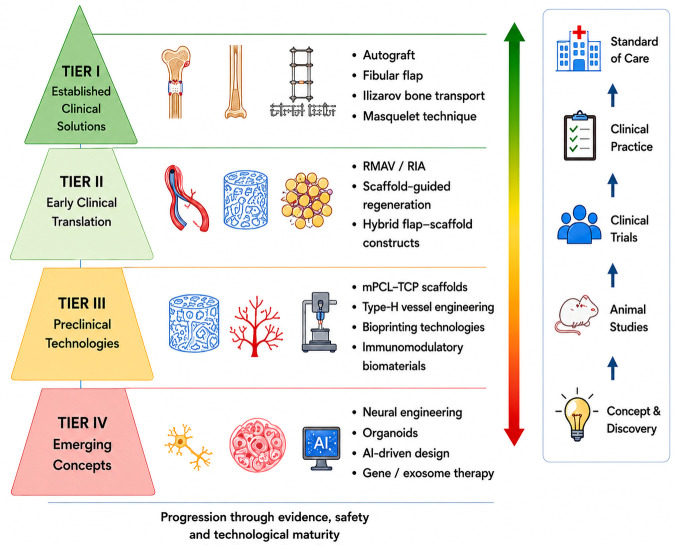
Translational pathway from concept to clinical practice. Hierarchical framework illustrating progression from emerging technologies through preclinical validation and early clinical translation to established reconstructive solutions.

These evidence gaps highlight priority areas for targeted investment, including adequately powered comparative trials, resolution of iPSC GMP and regulatory challenges, and the development of hybrid host–anastomosis strategies to address the bioprinting–capillary resolution mismatch. The relative translational maturity of current and emerging regenerative strategies is summarised in Figure 6.

## 9. Conclusions

The volumetric scaling problem in large bone defects involves challenges related to blood vessels, nerves, mechanics, the immune system, and the manufacturing process.

The principal contribution of this review is to articulate the volumetric scaling problem as a coupled, multi-system constraint that cannot be resolved by optimising any single component. Addressing it will require constructs that simultaneously achieve adequate vascularisation kinetics, mechanical load sharing, immune compatibility, and infection control, within the limits of patient-specific anatomy, regulatory requirements, and clinical timelines.

Current composite scaffolds are much less stiff than cortical bones and often require additional fixation during healing. The most robust large-animal data come from sheep tibial defects measuring 3 cm and 4 cm, whereas preclinical RMAV studies examine defects measuring 9.5 cm^3^ and 19 cm^3^. In the literature, a 36 cm human tibial reconstruction using RMAV and scaffold-guided regeneration is one of the largest reported examples of this approach for human long-bone reconstruction [58,59,60]. However, this case should be considered a rare, author-reported event in early first-in-human research, not an established standard of care.

This review highlights three main research priorities: First, different hybrid vascularisation strategies need to be directly compared in large-animal trials with sufficient power, since current evidence does not show which is best at the centimetre scale. Second, the best cell source for centimetre-scale reconstruction—whether expanded BM-MSCs, ASCs, lineage-restricted hSSCs, or iPSC-derived osteoprogenitors—should be identified through direct clinical comparisons rather than preclinical studies. Third, because current bioprinting cannot match the fine size of natural capillaries, hybrid host–anastomosis strategies need to be improved. In these approaches, printed channels support blood flow at the arteriolar level, and natural capillary networks are formed by the host after implantation.

Until these research priorities are addressed in well-designed comparative studies, established clinical options such as autograft, vascularised fibular transfer, distraction osteogenesis, and the Masquelet-induced membrane technique will remain the standard of care for centimetre-scale reconstruction.

## Figures and Tables

**Table 1:** Established clinical options for massive bone defects.

Approach	Mechanism	Practical Volumetric Limit	Principal Limitations
Iliac crest autograft	Osteogenic cells + osteoinductive factors + osteoconductive matrix [2]	Finite, patient-dependent graft volume (commonly tens of millilitres per donor site)	Donor-site morbidity; finite supply; lacks structural strength as an unsupported graft in load-bearing diaphyses
Structural cortical allograft	Cortical strut or segment; slow and often incomplete creeping substitution [6,7]	Longer segments available; practical limit depends on site, fixation, and host biology	Host–graft non-union; late fatigue fracture; mechanical deterioration [7]; infection; immunogenicity [8,9]
Distraction osteogenesis (Ilizarov)	Gradual mechanical lengthening drives endogenous bone formation in the regenerate	Large defects, commonly 6–10 cm; longer defects are possible in selected cases	Prolonged fixation; pin-site complications; regenerate consolidation problems; docking-site delayed union or non-union; high patient burden
Vascularised fibular flap	Microsurgical transfer of living bone with intrinsic blood supply	Long vascularised cortical segment; length depends on patient anatomy and donor-site constraints	Donor-site morbidity; tubular geometry; limited initial mechanical strength until union, and adaptive hypertrophy
Masquelet-induced membrane	PMMA spacer induces vascular, growth factor-rich biological membrane containing subsequent cancellous autograft [14]	Reported in very large defects (>15 cm in case reports/series)	Two-stage procedure; complication risk; requires sufficient graft volume, stable fixation, host biology, and soft-tissue coverage
Custom patient-specific metallic implant/tumour endoprosthesis	3D-printed titanium cage, modular tumour prosthesis, or reconstruction nail with cage; space-filling rather than regenerative; permanent implant [2]	Oncological or post-traumatic diaphyseal and periarticular defects; large segments possible with patient-specific CAD/CAM design	No bone regeneration; aseptic loosening; periprosthetic fracture; infection; revision complexity; lacks biological remodelling capacity

**Table 2:** Biological and clinical constraints across common massive bone-defect reconstruction contexts.

Defect Context	Loading Environment	Vascular Bed	Soft-Tissue Envelope	Infection Risk	Typical Fixation	Likely Regulatory Pathway
Long-bone diaphysis (post-traumatic)	Axial, bending, torsion	Periosteal + endosteal	Often compromised	Moderate–high	IM nail or plate	Device or combination product
Long-bone diaphysis (oncological)	Full weight-bearing	Usually preserved	Usually intact	Low–moderate	IM nail or plate	Combination product/ATMP
Mandible (oncological/post-traumatic)	Masticatory; non-weight-bearing	Terminal, radiation-sensitive	Frequently compromised	Moderate (oral flora)	Titanium reconstruction plate	Combination product/ATMP
Infected/osteomyelitis-related	Full weight-bearing	Compromised	Scarred/fistulated	Very high	Staged; external fixation common	ATMP + infection-control pathway

ATMP, advanced therapy medicinal product; IM, intramedullary. Categories are indicative generalisations; individual cases vary with anatomy, host biology, and jurisdiction.

**Table 4:** Selected large-animal and human studies of scaffold-based reconstruction of segmental long-bone defects.

Study	Defect Model and Size	Construct	Follow-Up	Comparator and Principal Finding
Reichert 2012 [34] (Preclinical; large-animal model)	Sheep tibia, 3 cm mid-diaphyseal	mPCL-TCP + rhBMP-7 (3.5 mg) vs. mPCL-TCP + BM-MSCs vs. mPCL-TCP alone	12 months	rhBMP-7 + scaffold performed at least comparably with autograft; scaffold alone and BM-MSC/scaffold groups were inferior
Cipitria 2013 [35] (Preclinical; large-animal model)	Sheep tibia, 3 cm mid-diaphyseal	mPCL-TCP + rhBMP-7 at low (1.75 mg) vs. high (3.5 mg) dose	12 months	Reduced-dose rhBMP-7 produced outcomes comparable with the higher dose in that model
Pobloth 2018 [28] (Preclinical; large-animal model)	Sheep tibia, approximately 4 cm segmental	Mechanobiologically optimised additively manufactured Ti-mesh, two strut thicknesses (high vs. low effective stiffness)	24 weeks	Lower-stiffness, less stress-shielded scaffold produced earlier defect bridging and greater endochondral bone formation
Petite 2000 [57] (Preclinical; large-animal model)	Sheep metatarsus segmental defect	Coralline scaffold (calcium carbonate) + BM-MSCs vs. scaffold alone vs. fresh marrow	16 weeks	Scaffold + expanded BM-MSCs achieved superior bone formation compared with scaffold alone or fresh marrow in that model
Sparks 2023 (preclinical) [58] (Preclinical; large-animal model)	Sheep tibia, 3 cm M-size (9.5 cm^3^); XL pilot 6 cm (19 cm^3^)	mPCL-TCP + autologous corticoperiosteal flap (RMAV; no exogenous rhBMP—biological component provided by autologous corticoperiosteal tissue)	12 months	RMAV comparable to autograft and superior to scaffold alone in the reported study; XL pilot provided preliminary scalability data
Sparks 2023/Castrisos 2022 [58,59] (First-in-human series; case-report level)	Human tibia, 36 cm intercalary defect after osteomyelitis in a 27-year-old male	mPCL-TCP + corticoperiosteal flap (RMAV; no exogenous rhBMP)	Multi-year follow-up reported	Weight-bearing radiographic consolidation reported by the authors; case-report level evidence

## Data Availability

The data supporting the findings of this study are available within the article. No additional datasets were generated or analysed beyond those included in this publication.

## Abbreviations

The following abbreviations are used in this manuscript: ASC: adipose-derived stromal cell; β-TCP: beta-tricalcium phosphate; BCP: biphasic calcium phosphate; BM-MSC: bone marrow-derived mesenchymal stromal cell; BMP-2: bone morphogenetic protein-2; CAT: Committee for Advanced Therapies (EMA); CBER: Center for Biologics Evaluation and Research; CGRP: calcitonin gene-related peptide; EMA: European Medicines Agency; FDA: United States Food and Drug Administration; GMP: good manufacturing practice; HA: hydroxyapatite; hSSC: human skeletal stem cell; iPSC: induced pluripotent stem cell; IQR: interquartile range; ITOP: integrated tissue–organ printer; mPCL-TCP: medical-grade polycaprolactone/β-tricalcium phosphate; MSC: mesenchymal stromal cell; NGF: nerve growth factor; OTP: Office of Therapeutic Products (FDA CBER); PCL: polycaprolactone; PDGF-BB: platelet-derived growth factor BB; PLA: polylactic acid; PLGA: poly(lactic-co-glycolic acid); PMMA: polymethylmethacrylate; PVDF: polyvinylidene fluoride; rhBMP-7: recombinant human bone morphogenetic protein-7; RMAV: regenerative matching axial vascularisation; TPMS: triply periodic minimal surface; VEGF: vascular endothelial growth factor.
